# (2*E*)-3-(4-Methyl­phen­yl)-1-(2-methyl-4-phenyl­quinolin-3-yl)prop-2-en-1-one monohydrate

**DOI:** 10.1107/S1600536810038791

**Published:** 2010-10-02

**Authors:** R. Prasath, S. Sarveswari, V. Vijayakumar, Seik Weng Ng, Edward R. T. Tiekink

**Affiliations:** a Organic Chemistry Division, School of Advanced Sciences, VIT University, Vellore 632 014, India; b Department of Chemistry, University of Malaya, 50603 Kuala Lumpur, Malaysia

## Abstract

The title hydrate, C_26_H_21_NO·H_2_O, exhibits significant twists of the benzene ring [dihedral angle = 87.24 (6)°] and chalcone residue [C—C—C—C torsion angle = −94.46 (17)°] out of the plane through the quinoline ring system. The conformation about the C=C bond [1.341 (2) Å] is *E*. The solvent water mol­ecule forms hydrogen bonds to carbonyl O and quinoline N atoms derived from two mol­ecules and through the application of a centre of inversion, a 16-membered {⋯HOH⋯OC_3_N}_2_ synthon is formed to stabilize the resulting tetra­meric (two organic mol­ecules plus two water mol­ecules) aggregate. These are connected into a two-dimensional array *via* two C—H⋯O contacts, also involving the water mol­ecule. The layers stack along the *c* axis, being linked by C—H⋯π inter­actions.

## Related literature

For background to chalcones, see: Prasath *et al.* (2010 ▶); Roman (2004 ▶).
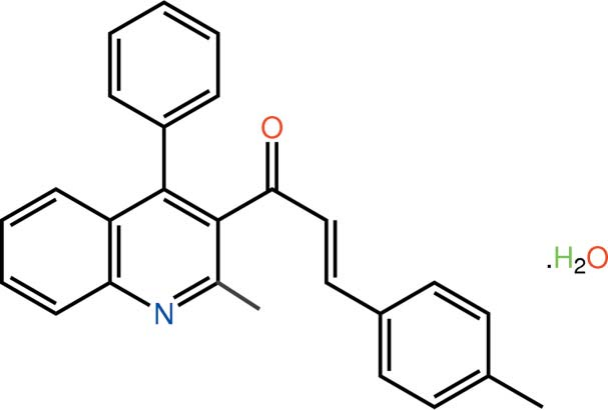


## Experimental

### 

#### Crystal data

C_26_H_21_NO·H_2_O
*M* *_r_* = 381.45
Triclinic, 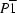

*a* = 8.2634 (7) Å
*b* = 9.0785 (7) Å
*c* = 14.1176 (12) Å
α = 91.137 (1)°
β = 101.537 (1)°
γ = 100.820 (1)°
*V* = 1017.43 (15) Å^3^
*Z* = 2
Mo *K*α radiation
μ = 0.08 mm^−1^
*T* = 100 K
0.30 × 0.25 × 0.20 mm

#### Data collection

Bruker SMART APEX CCD diffractometer
Absorption correction: multi-scan (*SADABS*; Sheldrick, 1996 ▶) *T*_min_ = 0.794, *T*_max_ = 0.862
9738 measured reflections
4647 independent reflections
3790 reflections with *I* > 2σ(*I*)
*R*_int_ = 0.023

#### Refinement

*R*[*F* ^2^ > 2σ(*F* ^2^)] = 0.046
*wR*(*F* ^2^) = 0.165
*S* = 1.01
4647 reflections
270 parameters
3 restraints
H atoms treated by a mixture of independent and constrained refinement
Δρ_max_ = 0.35 e Å^−3^
Δρ_min_ = −0.27 e Å^−3^

### 

Data collection: *APEX2* (Bruker, 2008 ▶); cell refinement: *SAINT* (Bruker, 2008 ▶); data reduction: *SAINT*; program(s) used to solve structure: *SHELXS97* (Sheldrick, 2008 ▶); program(s) used to refine structure: *SHELXL97* (Sheldrick, 2008 ▶); molecular graphics: *ORTEP-3* (Farrugia, 1997 ▶) and *DIAMOND* (Brandenburg, 2006 ▶); software used to prepare material for publication: *publCIF* (Westrip, 2010 ▶).

## Supplementary Material

Crystal structure: contains datablocks global, I. DOI: 10.1107/S1600536810038791/hb5655sup1.cif

Structure factors: contains datablocks I. DOI: 10.1107/S1600536810038791/hb5655Isup2.hkl

Additional supplementary materials:  crystallographic information; 3D view; checkCIF report

## Figures and Tables

**Table 1:** Hydrogen-bond geometry (Å, °) *Cg*1 is the centroid of the C11–C16 ring.

*D*—H⋯*A*	*D*—H	H⋯*A*	*D*⋯*A*	*D*—H⋯*A*
O1w—H1w⋯O1	0.85 (1)	2.03 (1)	2.8654 (17)	166 (2)
O1w—H2w⋯N1^i^	0.85 (1)	2.06 (1)	2.9032 (17)	170 (2)
C4—H4⋯O1w^ii^	0.95	2.49	3.4055 (19)	161
C16—H16⋯O1w^iii^	0.95	2.52	3.402 (2)	155
C24—H24⋯*Cg*1^iv^	0.95	2.70	3.6414 (17)	171

Symmetry codes: (i) 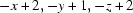; (ii) 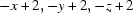; (iii) 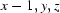; (iv) 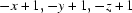.

**Fig. 1.**
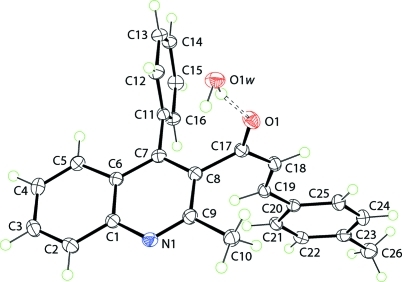
Molecular structure of (I) showing displacement ellipsoids at the 50% probability level. The O—H···O hydrogen bond is shown as a dashed line.

**Fig. 2.**
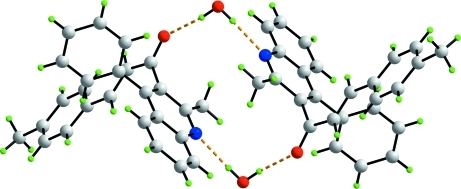
Tetrameric aggregate in (I) sustained by O—H···O, N hydrogen bonds shown as orange dashed lines.

**Fig. 3.**
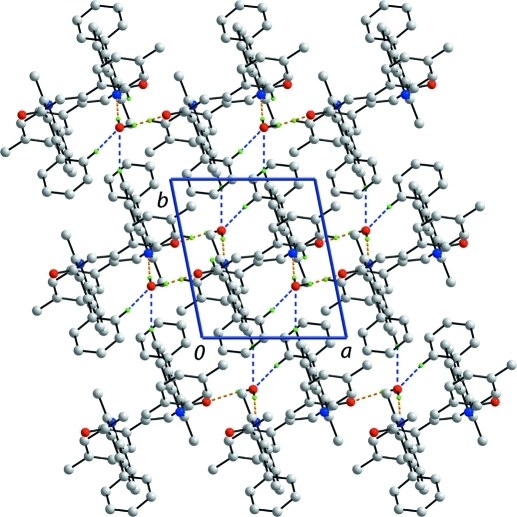
Supramolecular array in the ab plane. The O—H···O, N hydrogen bonds and C—H···O interactions are shown as orange and blue dashed lines, respectively. Hydrogen atoms not participating in interactions to stabilize the array have been omitted for clarity.

**Fig. 4.**
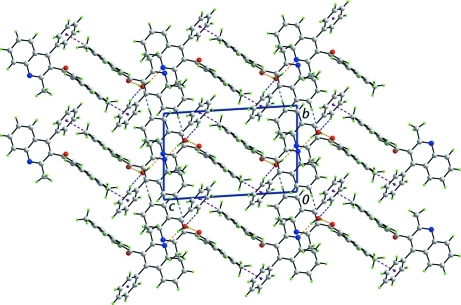
Unit-cell contents for (I) viewed in projection along the a axis showing the stacking of layers along the c axis. The O—H···O, N hydrogen bonds and C—H···O and C—H···π contacts are shown as orange, blue and purple dashed lines, respectively.

## supplementary crystallographic information

### Comment

Chalcones and their corresponding heterocyclic analogues are valuable intermediates in organic synthesis and exhibit a multitude of biological activities. From a chemical point of view, an important feature of chalcones and their analogues is their ability to act as activated unsaturated systems in conjugated addition reactions of carbanions in the presence of basic catalysts (Roman, 2004). In continuation of our interest in the synthesis and crystallographic analysis of chalcones (Prasath *et al.*, 2010), herein we report the structure of a new chalcone derivative isolated as an hydrate, (I).

With reference to the quinolinyl residue (r.m.s. deviation = 0.014 Å), the benzene ring is almost normal, forming a dihedral angle of 87.24 (6) °. The chalcone residue also occupies a position normal to the quinolinyl group as seen in the value of the C7—C8—C17—C18 torsion angle of -94.46 (17) °. The conformation about the C18═C19 double bond [1.341 (2) Å] is *E*. Small twists are seen in the 4-methylphenyl)prop-2-en-1-one group so that while the expected planar arrangement is seen around the double bond [C17—C18—C19—C20 = -177.71 (13) °], the terminal benzene ring is twisted out of the plane [C18—C19—C20—C21 = -166.79 (15) °].

As anticipated, the water molecule plays a pivotal role in arranging molecules in the crystal packing. Two centrosymmetrically related water molecules link two organic molecules by forming hydrogen bonds to carbonyl-O and quinolinyl-N atoms, Table 1. In this way a 16-membered {···HOH···OC_3_N}_2_ synthon is formed, Fig. 2. These are connected into a supramolecular layer in the *ab* plane *via* two C—H···O contacts where the water-O accepts these interactions, Fig. 3 and Table 1. Layers stack along the *c* axis, being connected by C—H···π interactions, Fig. 4 and Table 1.

### Experimental

A mixture of 3-acetyl-2-methyl-4-phenylquinoline (2.6 g, 0.01 mmol), 4-methylbenzaldehyde (1.2 g, 0.01 mmol) and a catalytic amount of KOH was stirred in distilled ethanol (25 ml) for about 12 h. The resulting mixture was concentrated to remove ethanol, then poured on to ice and neutralized with dilute acetic acid. The resultant solid was filtered, dried and purified by column chromatography using an 1:1 mixture of ethyl acetate and petroleum ether. Re-crystallization was by slow evaporation of an acetone solution of (I) which yielded colourless crystals; Yield: 64%, *M*.pt. 412–414 K.

### Refinement

Carbon-bound H-atoms were placed in calculated positions (C—H 0.95 to 0.98 Å) and were included in the refinement in the riding model approximation, with *U*_iso_(H) set to 1.2 to 1.5*U*_equiv_(C). The O-bound H atoms were refined with the distance restraint O—H = 0.84±0.1 Å, and with *U*_iso_(H) = 1.5*U*_equiv_(O). In the final refinement a low angle reflection evidently effected by the beam stop was omitted, *i.e*. (0 0 1).

### Crystal data

**Table:**

C_26_H_21_NO·H_2_O	*Z* = 2
*M**_r_* = 381.45	*F*(000) = 404
Triclinic, *P*1	*D*_x_ = 1.245 Mg m^−^^3^
Hall symbol: -P 1	Mo *K*α radiation, λ = 0.71073 Å
*a* = 8.2634 (7) Å	Cell parameters from 4003 reflections
*b* = 9.0785 (7) Å	θ = 2.3–28.3°
*c* = 14.1176 (12) Å	µ = 0.08 mm^−^^1^
α = 91.137 (1)°	*T* = 100 K
β = 101.537 (1)°	Block, colourless
γ = 100.820 (1)°	0.30 × 0.25 × 0.20 mm
*V* = 1017.43 (15) Å^3^	

### Data collection

**Table:**

Bruker SMART APEX CCD diffractometer	4647 independent reflections
Radiation source: fine-focus sealed tube	3790 reflections with *I* > 2σ(*I*)
graphite	*R*_int_ = 0.023
ω scans	θ_max_ = 27.5°, θ_min_ = 2.3°
Absorption correction: multi-scan (*SADABS*; Sheldrick, 1996)	*h* = −10→10
*T*_min_ = 0.794, *T*_max_ = 0.862	*k* = −11→11
9738 measured reflections	*l* = −18→18

### Refinement

**Table:**

Refinement on *F*^2^	Primary atom site location: structure-invariant direct methods
Least-squares matrix: full	Secondary atom site location: difference Fourier map
*R*[*F*^2^ > 2σ(*F*^2^)] = 0.046	Hydrogen site location: inferred from neighbouring sites
*wR*(*F*^2^) = 0.165	H atoms treated by a mixture of independent and constrained refinement
*S* = 1.01	*w* = 1/[σ^2^(*F*_o_^2^) + (0.1041*P*)^2^ + 0.3702*P*] where *P* = (*F*_o_^2^ + 2*F*_c_^2^)/3
4647 reflections	(Δ/σ)_max_ = 0.001
270 parameters	Δρ_max_ = 0.35 e Å^−^^3^
3 restraints	Δρ_min_ = −0.27 e Å^−^^3^

### Special details

**Table:**

Geometry. All s.u.'s (except the s.u. in the dihedral angle between two l.s. planes) are estimated using the full covariance matrix. The cell s.u.'s are taken into account individually in the estimation of s.u.'s in distances, angles and torsion angles; correlations between s.u.'s in cell parameters are only used when they are defined by crystal symmetry. An approximate (isotropic) treatment of cell s.u.'s is used for estimating s.u.'s involving l.s. planes.
Refinement. Refinement of *F*^2^ against ALL reflections. The weighted *R*-factor *wR* and goodness of fit *S* are based on *F*^2^, conventional *R*-factors *R* are based on *F*, with *F* set to zero for negative *F*^2^. The threshold expression of *F*^2^ > 2σ(*F*^2^) is used only for calculating *R*-factors(gt) *etc*. and is not relevant to the choice of reflections for refinement. *R*-factors based on *F*^2^ are statistically about twice as large as those based on *F*, and *R*- factors based on ALL data will be even larger.

### Fractional atomic coordinates and isotropic or equivalent isotropic displacement parameters (Å^2^)

**Table:**

	*x*	*y*	*z*	*U*_iso_*/*U*_eq_	
O1	0.94050 (14)	0.59600 (13)	0.73474 (8)	0.0254 (3)	
O1W	1.28132 (15)	0.67478 (13)	0.84124 (8)	0.0250 (3)	
H1W	1.1758 (13)	0.660 (2)	0.8172 (15)	0.037*	
H2W	1.280 (3)	0.623 (2)	0.8910 (11)	0.037*	
N1	0.74421 (16)	0.53267 (14)	1.00612 (9)	0.0196 (3)	
C1	0.71971 (18)	0.67293 (16)	1.03036 (10)	0.0177 (3)	
C2	0.6977 (2)	0.70135 (18)	1.12519 (11)	0.0224 (3)	
H2	0.6987	0.6239	1.1695	0.027*	
C3	0.6751 (2)	0.83978 (18)	1.15372 (11)	0.0232 (3)	
H3	0.6601	0.8577	1.2176	0.028*	
C4	0.67398 (19)	0.95613 (17)	1.08882 (11)	0.0227 (3)	
H4	0.6585	1.0519	1.1092	0.027*	
C5	0.69517 (19)	0.93110 (17)	0.99649 (11)	0.0196 (3)	
H5	0.6947	1.0101	0.9533	0.024*	
C6	0.71780 (17)	0.78851 (16)	0.96457 (10)	0.0169 (3)	
C7	0.73952 (17)	0.75512 (16)	0.86916 (10)	0.0166 (3)	
C8	0.76569 (18)	0.61495 (16)	0.84716 (10)	0.0169 (3)	
C9	0.76789 (18)	0.50488 (17)	0.91867 (11)	0.0190 (3)	
C10	0.8000 (2)	0.35126 (17)	0.89604 (12)	0.0245 (3)	
H10A	0.8047	0.2937	0.9542	0.037*	
H10B	0.9073	0.3616	0.8749	0.037*	
H10C	0.7087	0.2987	0.8443	0.037*	
C11	0.73297 (18)	0.87247 (16)	0.79625 (10)	0.0173 (3)	
C12	0.88036 (19)	0.96807 (17)	0.78550 (11)	0.0214 (3)	
H12	0.9857	0.9572	0.8235	0.026*	
C13	0.8738 (2)	1.07962 (17)	0.71923 (12)	0.0232 (3)	
H13	0.9745	1.1449	0.7123	0.028*	
C14	0.7202 (2)	1.09540 (17)	0.66338 (11)	0.0218 (3)	
H14	0.7157	1.1709	0.6178	0.026*	
C15	0.5731 (2)	1.00064 (17)	0.67422 (11)	0.0225 (3)	
H15	0.4680	1.0117	0.6360	0.027*	
C16	0.57866 (19)	0.88978 (17)	0.74058 (11)	0.0197 (3)	
H16	0.4775	0.8258	0.7481	0.024*	
C17	0.79675 (18)	0.57226 (16)	0.74902 (11)	0.0179 (3)	
C18	0.65484 (19)	0.49748 (16)	0.67431 (10)	0.0189 (3)	
H18	0.6776	0.4576	0.6167	0.023*	
C19	0.49390 (19)	0.48223 (16)	0.68281 (10)	0.0181 (3)	
H19	0.4744	0.5264	0.7400	0.022*	
C20	0.34572 (18)	0.40472 (16)	0.61317 (10)	0.0177 (3)	
C21	0.18670 (19)	0.42552 (17)	0.62322 (11)	0.0203 (3)	
H21	0.1774	0.4912	0.6742	0.024*	
C22	0.04245 (19)	0.35188 (17)	0.56003 (11)	0.0215 (3)	
H22	−0.0642	0.3687	0.5680	0.026*	
C23	0.0509 (2)	0.25349 (17)	0.48501 (11)	0.0221 (3)	
C24	0.2101 (2)	0.23307 (18)	0.47450 (11)	0.0227 (3)	
H24	0.2189	0.1672	0.4235	0.027*	
C25	0.3546 (2)	0.30677 (17)	0.53679 (11)	0.0216 (3)	
H25	0.4613	0.2912	0.5280	0.026*	
C26	−0.1064 (2)	0.1710 (2)	0.41757 (12)	0.0291 (4)	
H26A	−0.2011	0.2191	0.4232	0.044*	
H26B	−0.1308	0.0663	0.4348	0.044*	
H26C	−0.0898	0.1740	0.3508	0.044*	

### Atomic displacement parameters (Å^2^)

**Table:**

	*U*^11^	*U*^22^	*U*^33^	*U*^12^	*U*^13^	*U*^23^
O1	0.0202 (6)	0.0342 (6)	0.0227 (6)	0.0050 (5)	0.0068 (4)	0.0002 (5)
O1W	0.0252 (6)	0.0248 (6)	0.0232 (6)	0.0007 (5)	0.0042 (5)	0.0061 (5)
N1	0.0217 (6)	0.0181 (6)	0.0185 (6)	0.0030 (5)	0.0040 (5)	0.0034 (5)
C1	0.0155 (7)	0.0191 (7)	0.0176 (7)	0.0021 (5)	0.0027 (5)	0.0008 (5)
C2	0.0230 (8)	0.0263 (8)	0.0182 (7)	0.0046 (6)	0.0050 (6)	0.0045 (6)
C3	0.0219 (7)	0.0295 (8)	0.0183 (7)	0.0039 (6)	0.0058 (6)	−0.0025 (6)
C4	0.0209 (7)	0.0224 (7)	0.0238 (8)	0.0052 (6)	0.0022 (6)	−0.0046 (6)
C5	0.0190 (7)	0.0184 (7)	0.0209 (7)	0.0040 (5)	0.0027 (6)	0.0013 (6)
C6	0.0143 (6)	0.0183 (7)	0.0169 (7)	0.0021 (5)	0.0013 (5)	0.0006 (5)
C7	0.0120 (6)	0.0180 (7)	0.0184 (7)	0.0015 (5)	0.0013 (5)	0.0017 (5)
C8	0.0145 (6)	0.0191 (7)	0.0162 (7)	0.0020 (5)	0.0021 (5)	0.0007 (5)
C9	0.0187 (7)	0.0185 (7)	0.0184 (7)	0.0029 (5)	0.0015 (5)	0.0004 (5)
C10	0.0328 (9)	0.0195 (7)	0.0225 (8)	0.0081 (6)	0.0056 (6)	0.0026 (6)
C11	0.0203 (7)	0.0168 (7)	0.0150 (7)	0.0044 (5)	0.0039 (5)	0.0007 (5)
C12	0.0169 (7)	0.0242 (7)	0.0208 (7)	0.0013 (6)	0.0010 (6)	0.0024 (6)
C13	0.0230 (8)	0.0205 (7)	0.0245 (8)	−0.0021 (6)	0.0071 (6)	0.0021 (6)
C14	0.0287 (8)	0.0177 (7)	0.0204 (7)	0.0054 (6)	0.0072 (6)	0.0053 (6)
C15	0.0203 (7)	0.0243 (8)	0.0231 (8)	0.0069 (6)	0.0020 (6)	0.0039 (6)
C16	0.0166 (7)	0.0205 (7)	0.0217 (7)	0.0024 (5)	0.0042 (6)	0.0032 (6)
C17	0.0196 (7)	0.0165 (7)	0.0190 (7)	0.0055 (5)	0.0053 (6)	0.0027 (5)
C18	0.0230 (7)	0.0190 (7)	0.0156 (7)	0.0053 (6)	0.0047 (6)	0.0005 (5)
C19	0.0221 (7)	0.0184 (7)	0.0146 (7)	0.0054 (6)	0.0040 (6)	0.0008 (5)
C20	0.0204 (7)	0.0176 (7)	0.0157 (7)	0.0042 (5)	0.0042 (5)	0.0036 (5)
C21	0.0233 (8)	0.0210 (7)	0.0186 (7)	0.0054 (6)	0.0077 (6)	0.0021 (6)
C22	0.0207 (7)	0.0242 (7)	0.0213 (7)	0.0044 (6)	0.0077 (6)	0.0058 (6)
C23	0.0228 (8)	0.0212 (7)	0.0204 (7)	0.0007 (6)	0.0034 (6)	0.0047 (6)
C24	0.0250 (8)	0.0237 (7)	0.0186 (7)	0.0046 (6)	0.0035 (6)	−0.0024 (6)
C25	0.0211 (7)	0.0238 (7)	0.0211 (7)	0.0072 (6)	0.0047 (6)	−0.0005 (6)
C26	0.0249 (8)	0.0316 (9)	0.0265 (8)	−0.0012 (7)	0.0016 (7)	0.0009 (7)

### Geometric parameters (Å, °)

**Table:**

O1—C17	1.2245 (18)	C12—H12	0.9500
O1W—H1W	0.854 (10)	C13—C14	1.388 (2)
O1W—H2W	0.852 (9)	C13—H13	0.9500
N1—C9	1.3149 (19)	C14—C15	1.389 (2)
N1—C1	1.3736 (18)	C14—H14	0.9500
C1—C2	1.412 (2)	C15—C16	1.389 (2)
C1—C6	1.415 (2)	C15—H15	0.9500
C2—C3	1.369 (2)	C16—H16	0.9500
C2—H2	0.9500	C17—C18	1.458 (2)
C3—C4	1.412 (2)	C18—C19	1.341 (2)
C3—H3	0.9500	C18—H18	0.9500
C4—C5	1.370 (2)	C19—C20	1.459 (2)
C4—H4	0.9500	C19—H19	0.9500
C5—C6	1.4196 (19)	C20—C21	1.396 (2)
C5—H5	0.9500	C20—C25	1.406 (2)
C6—C7	1.428 (2)	C21—C22	1.384 (2)
C7—C8	1.370 (2)	C21—H21	0.9500
C7—C11	1.497 (2)	C22—C23	1.393 (2)
C8—C9	1.434 (2)	C22—H22	0.9500
C8—C17	1.5146 (19)	C23—C24	1.398 (2)
C9—C10	1.507 (2)	C23—C26	1.506 (2)
C10—H10A	0.9800	C24—C25	1.380 (2)
C10—H10B	0.9800	C24—H24	0.9500
C10—H10C	0.9800	C25—H25	0.9500
C11—C12	1.394 (2)	C26—H26A	0.9800
C11—C16	1.396 (2)	C26—H26B	0.9800
C12—C13	1.394 (2)	C26—H26C	0.9800
			
H1W—O1W—H2W	100 (2)	C12—C13—H13	120.0
C9—N1—C1	118.81 (13)	C13—C14—C15	119.89 (14)
N1—C1—C2	117.92 (13)	C13—C14—H14	120.1
N1—C1—C6	122.51 (13)	C15—C14—H14	120.1
C2—C1—C6	119.57 (13)	C14—C15—C16	120.39 (14)
C3—C2—C1	120.42 (15)	C14—C15—H15	119.8
C3—C2—H2	119.8	C16—C15—H15	119.8
C1—C2—H2	119.8	C15—C16—C11	119.95 (14)
C2—C3—C4	120.46 (14)	C15—C16—H16	120.0
C2—C3—H3	119.8	C11—C16—H16	120.0
C4—C3—H3	119.8	O1—C17—C18	121.12 (13)
C5—C4—C3	120.12 (14)	O1—C17—C8	119.76 (13)
C5—C4—H4	119.9	C18—C17—C8	119.07 (12)
C3—C4—H4	119.9	C19—C18—C17	123.29 (13)
C4—C5—C6	120.74 (14)	C19—C18—H18	118.4
C4—C5—H5	119.6	C17—C18—H18	118.4
C6—C5—H5	119.6	C18—C19—C20	126.63 (13)
C1—C6—C5	118.69 (13)	C18—C19—H19	116.7
C1—C6—C7	117.89 (13)	C20—C19—H19	116.7
C5—C6—C7	123.41 (13)	C21—C20—C25	117.89 (14)
C8—C7—C6	118.43 (13)	C21—C20—C19	119.03 (13)
C8—C7—C11	121.85 (13)	C25—C20—C19	123.06 (13)
C6—C7—C11	119.71 (12)	C22—C21—C20	121.01 (13)
C7—C8—C9	120.04 (13)	C22—C21—H21	119.5
C7—C8—C17	121.83 (13)	C20—C21—H21	119.5
C9—C8—C17	118.12 (12)	C21—C22—C23	121.16 (14)
N1—C9—C8	122.29 (13)	C21—C22—H22	119.4
N1—C9—C10	117.14 (13)	C23—C22—H22	119.4
C8—C9—C10	120.57 (13)	C22—C23—C24	117.93 (14)
C9—C10—H10A	109.5	C22—C23—C26	121.08 (14)
C9—C10—H10B	109.5	C24—C23—C26	120.99 (14)
H10A—C10—H10B	109.5	C25—C24—C23	121.28 (14)
C9—C10—H10C	109.5	C25—C24—H24	119.4
H10A—C10—H10C	109.5	C23—C24—H24	119.4
H10B—C10—H10C	109.5	C24—C25—C20	120.72 (14)
C12—C11—C16	119.54 (14)	C24—C25—H25	119.6
C12—C11—C7	120.23 (13)	C20—C25—H25	119.6
C16—C11—C7	120.21 (13)	C23—C26—H26A	109.5
C11—C12—C13	120.23 (14)	C23—C26—H26B	109.5
C11—C12—H12	119.9	H26A—C26—H26B	109.5
C13—C12—H12	119.9	C23—C26—H26C	109.5
C14—C13—C12	120.00 (14)	H26A—C26—H26C	109.5
C14—C13—H13	120.0	H26B—C26—H26C	109.5
			
C9—N1—C1—C2	−178.81 (14)	C8—C7—C11—C16	94.05 (17)
C9—N1—C1—C6	0.4 (2)	C6—C7—C11—C16	−85.84 (17)
N1—C1—C2—C3	179.12 (14)	C16—C11—C12—C13	−0.4 (2)
C6—C1—C2—C3	−0.2 (2)	C7—C11—C12—C13	−178.49 (13)
C1—C2—C3—C4	−0.2 (2)	C11—C12—C13—C14	−0.3 (2)
C2—C3—C4—C5	0.2 (2)	C12—C13—C14—C15	0.5 (2)
C3—C4—C5—C6	0.3 (2)	C13—C14—C15—C16	−0.1 (2)
N1—C1—C6—C5	−178.69 (13)	C14—C15—C16—C11	−0.5 (2)
C2—C1—C6—C5	0.6 (2)	C12—C11—C16—C15	0.7 (2)
N1—C1—C6—C7	1.1 (2)	C7—C11—C16—C15	178.87 (13)
C2—C1—C6—C7	−179.66 (13)	C7—C8—C17—O1	88.14 (18)
C4—C5—C6—C1	−0.6 (2)	C9—C8—C17—O1	−90.49 (17)
C4—C5—C6—C7	179.62 (14)	C7—C8—C17—C18	−94.46 (17)
C1—C6—C7—C8	−1.8 (2)	C9—C8—C17—C18	86.91 (17)
C5—C6—C7—C8	177.97 (13)	O1—C17—C18—C19	−173.19 (14)
C1—C6—C7—C11	178.09 (13)	C8—C17—C18—C19	9.4 (2)
C5—C6—C7—C11	−2.1 (2)	C17—C18—C19—C20	−177.71 (13)
C6—C7—C8—C9	1.1 (2)	C18—C19—C20—C21	−166.79 (15)
C11—C7—C8—C9	−178.82 (13)	C18—C19—C20—C25	14.5 (2)
C6—C7—C8—C17	−177.54 (13)	C25—C20—C21—C22	0.1 (2)
C11—C7—C8—C17	2.6 (2)	C19—C20—C21—C22	−178.68 (13)
C1—N1—C9—C8	−1.3 (2)	C20—C21—C22—C23	0.7 (2)
C1—N1—C9—C10	178.05 (13)	C21—C22—C23—C24	−1.0 (2)
C7—C8—C9—N1	0.5 (2)	C21—C22—C23—C26	178.73 (14)
C17—C8—C9—N1	179.17 (13)	C22—C23—C24—C25	0.6 (2)
C7—C8—C9—C10	−178.78 (14)	C26—C23—C24—C25	−179.16 (15)
C17—C8—C9—C10	−0.1 (2)	C23—C24—C25—C20	0.2 (2)
C8—C7—C11—C12	−87.84 (18)	C21—C20—C25—C24	−0.5 (2)
C6—C7—C11—C12	92.27 (17)	C19—C20—C25—C24	178.20 (14)

### Hydrogen-bond geometry (Å, °)

**Table:**

*D*—H···*A*	*D*—H	H···*A*	*D*···*A*	*D*—H···*A*
O1w—H1w···O1	0.854 (10)	2.029 (11)	2.8654 (17)	166 (2)
O1w—H2w···N1^i^	0.852 (9)	2.061 (10)	2.9032 (17)	170 (2)
C4—H4···O1w^ii^	0.95	2.49	3.4055 (19)	161
C16—H16···O1w^iii^	0.95	2.52	3.402 (2)	155
C24—H24···Cg1^iv^	0.95	2.70	3.6414 (17)	171

Symmetry codes: (i) −*x*+2, −*y*+1, −*z*+2; (ii) −*x*+2, −*y*+2, −*z*+2; (iii) *x*−1, *y*, *z*; (iv) −*x*+1, −*y*+1, −*z*+1.

## References

[bb1] Brandenburg, K. (2006). *DIAMOND* Crystal Impact GbR, Bonn, Germany.

[bb2] Bruker (2008). *APEX2* and *SAINT* Bruker AXS Inc., Madison, Wisconsin, USA.

[bb3] Farrugia, L. J. (1997). *J. Appl. Cryst.* **30**, 565.

[bb4] Prasath, R., Sarveswari, S., Vijayakumar, V., Narasimhamurthy, T. & Tiekink, E. R. T. (2010). *Acta Cryst.* E**66**, o1110. 10.1107/S1600536810013784 PMC2979249 21579162

[bb5] Roman, G. (2004). *Acta Chim. Slov.* **51**, 537–544.

[bb6] Sheldrick, G. M. (1996). *SADABS* University of Göttingen, Germany.

[bb7] Sheldrick, G. M. (2008). *Acta Cryst.* A**64**, 112–122. 10.1107/S0108767307043930 18156677

[bb8] Westrip, S. P. (2010). *J. Appl. Cryst.* **43**, 920–925.

